# Chronic Myelomonocytic Leukemia Patients With Lysozyme Nephropathy and Renal Infiltration Display Markers of Severe Disease

**DOI:** 10.1016/j.ekir.2023.09.005

**Published:** 2023-09-07

**Authors:** Marie-Camille Lafargue, Mickaël Bobot, Helmut G. Rennke, Marie Essig, Martin Carre, Lucile Mercadal, Jonathan Farhi, Hamza Sakhi, Thibault Comont, Léonard Golbin, Pierre Isnard, Jonathan Chemouny, Nathalie Cambier, Kamel Laribi, Umut Selamet, Leonardo V. Riella, Olivier Fain, Lionel Adès, Pierre Fenaux, Camille Cohen, Arsène Mekinian, MINHEMON, GFM and French VEXAS group, Marie-Camille Lafargue, Mickaël Bobot, Thibault Comont, Nathalie Cambier, Kamel Laribi, Olivier Fain, Lionel Adès, Pierre Fenaux, Arsène Mekinian, Thibault Comont, Nathalie Cambier, Kamel Laribi, Olivier Fain, Lionel Adès, Pierre Fenaux, Arsène Mekinian, Thibault Comont, Olivier Fain, Lionel Adès, Pierre Fenaux, Arsène Mekinian

**Affiliations:** 1 Department of Nephrology, Tenon’s Hospital, Assistance Publique-Hôpitaux de Paris, Université Paris Cité, Paris, France; 2 Centre de Néphrologie et Transplantation Rénale, AP-HM, Hôpital de la Conception, CHU de la Conception, 13005, Marseille, France; 3 Department of Pathology, Brigham and Women's Hospital, Harvard Medical School, Boston, Massachusetts, USA; 4 Department of Nephrology, Assistance Publique-Hôpitaux de Paris, Hôpital Ambroise Paré, Boulogne-Billancourt, Université Paris-Saclay, Paris, France; 5 Service d'Hématologie, Centre Hospitalier Universitaire, Grenoble, France; 6 Department of Nephrology and Renal Transplantation, Assistance Publique-Hôpitaux de Paris, Hôpital de La Pitié Salpêtrière, Paris, France; 7 Maladies du Sang, CHU d'Angers, 49000 Angers, France; 8 Department of Nephrology and renal transplantation, Necker’s Hospital, Assistance Publique-Hôpitaux de Paris, Université Paris Cité, Paris, France; 9 Department of Internal Medicine, Institut universitaire du cancer de Toulouse, Centre Hospitalier Universitaire de Toulouse, Université Toulouse III - Paul Sabatier, Toulouse, France; 10 Service de Néphrologie, Dialyse et Transplantation Rénale, CHU Rennes, Hôpital Pontchaillou, Rennes, France; 11 Department of Pathology, Université Paris Cité, Necker Hospital, Assistance Publique-Hôpitaux de Paris, Paris, France; 12 Hématologie Clinique, Centre Hospitalier de Valenciennes, Valenciennes, France; 13 Department of Haematology, Centre Hospitalier Le Mans, France; 14 Centre Hospitalier Le Mans, Le Mans, France; 15 Division of Renal Medicine, Brigham and Women's Hospital, Boston, Massachusetts, USA; 16 Center of Transplantation Sciences, Department of Medicine, Division of Nephrology, Massachusetts General Hospital, Harvard Medical School, Charlestown, MA, United States; 17 Hôpital Saint-Antoine, Service de Médecine Interne et de l'Inflammation-(DHU i2B), F-75012, Assistance Publique-Hôpitaux de Paris, Paris, France; 18 Hématologie, Hôpital Saint-Louis, Assistance Publique-Hôpitaux de Paris, Université Paris Diderot, Paris, France; 19 INSERM U1151 « mechanisms and therapeutic strategies of chronic kidney diseases », Hôpital Necker, Université Paris Cité, Paris, France; Service de Néphrologie et Transplantation rénale, Assistance Publique-Hôpitaux de Paris, Hôpital Necker, Paris, France

**Keywords:** acute kidney injury, chronic myelomonocytic leukemia

## Abstract

**Introduction:**

Chronic myelomonocytic leukemia (CMML) is a hematologic disorder that is an overlap syndrome between myelodysplastic syndromes and myeloproliferative neoplasms, and can be associated with autoimmune and inflammatory diseases. This study aimed to describe kidney involvement in patients with CMML, their treatments, and outcomes.

**Methods:**

We conducted a French and American multicenter retrospective study in 15 centers, identifying patients with CMML with acute kidney injury (AKI), chronic kidney disease (CKD), and urine abnormalities.

**Results:**

Sixteen patients (males, *n* = 14; median age 76.5 years [71.9–83]) developed a kidney disease 6 months [1.6–25.6] after the diagnosis of CMML. At the time of kidney disease diagnosis, median urinary protein-to-creatinine ratio was 2 g/g [1.25–3.4], and median serum creatinine was 2.26 mg/dl [1.46–2.68]. Fourteen patients (87.5%) underwent a kidney biopsy, and the 2 main pathological findings were lysozyme nephropathy (56%) and renal infiltration by the CMML (37.5%). Ten patients received a new treatment following the CMML-associated kidney injury. Among patients with monitored kidney function, and after a median follow-up of 15 months [9.9–34.9], 4 patients had CKD stage 3, 4 had CKD stage 4, 1 had an end-stage kidney disease. In our patient series, 2 patients evolved to an acute myeloid leukemia (AML), and 5 died. Compared with 116 CMML controls, patients who had a kidney involvement had a higher monocyte count (*P* < 0.001), had more CMML-1 (*P* = 0.005), were more susceptible to develop an AML (*P* = 0.02), and were more eligible to receive a specific hematologic treatment, with hydroxyurea, or hypomethylating agents (*P* < 0.001), but no survival difference was seen between the 2 groups (*P* = 0.6978).

**Conclusion:**

In this cohort of patients with CMML with a kidney injury, the 2 most frequent renal complications were lysozyme-induced nephropathy and renal infiltration by the CMML. Kidney involvement should be closely monitored in patients with CMML.

## Graphical abstract

**Figure:**
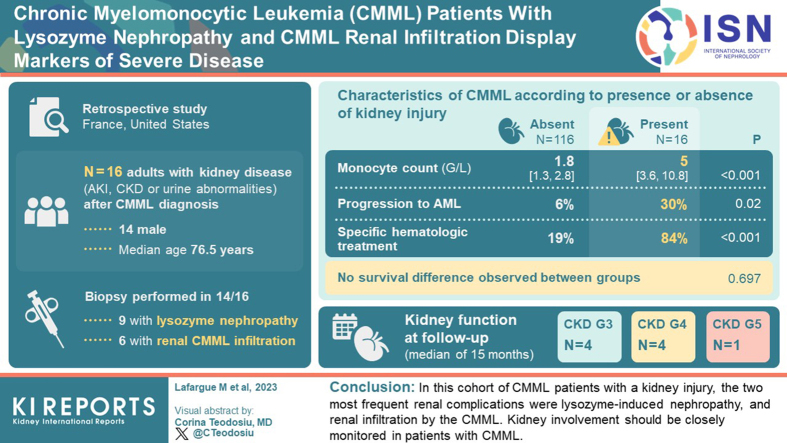

Kidney diseases in patients with hematological malignancies are common, with various etiologies.^1^ The development of kidney disease significantly worsens the prognosis of these patients. In the past few years, the concept of monoclonal gammopathy of renal significance has allowed the better characterization of kidney diseases related to monoclonal gammopathies.^2^ Nevertheless, renal complications associated with myeloid neoplasms are much less described.

CMML is an overlap syndrome that has characteristics of a myelodysplastic syndrome, whereas other findings are more consistent with the diagnosis of myeloproliferative neoplasm.^3^ CMML is characterized by persistent monocytosis, hypercellular marrow with granuloblastic hyperplasia, variable degrees of dysplasia, and increased monocytic cells. The diagnosis, prognostic, and therapeut management of CMML have been modified by the identification of molecular abnormalities.^4^

CMML can be associated with systemic inflammatory and autoimmune diseases in 10% to 30% of patients, mostly neutrophilic diseases, connective tissue disorders, arthritis, and vasculitis.^5^, ^6^, ^7^ The incidence of AKI and CKD is particularly high in patients with CMML (34.9%, and 7.6%, respectively).^8^ The landscape of kidney disorders in CMML is based on single-case studies and small cohorts. Several case reports suggest a relation between lysozyme-induced kidney injury and CMML.^9^, ^10^, ^11^, ^12^ Glomerular injury description remains elusive. Kidney injuries related to CMML were described in small cohorts, generally associated with myeloproliferative neoplasms.^13^^,^^14^ There is a higher incidence of AKI (34.9%) and CKD (7.6%) among patients with myeloproliferative form of CMML.^8^ Main findings revealed that tubulointerstitial compartment was the first kidney injury in a study of 8 CMML cases, with chronic tubulointerstitial nephropathy, and acute interstitial nephropathy.^13^ An interstitial infiltration can be seen, by T cells, or a massive infiltration by the CMML. Another histologic study of 4 patients showed glomerular injuries such as endothelial damage, mesangiolysis, double contours, and rare lesions of podocytopathy and thrombotic microangiopathy.^14^ Nevertheless, the size of these cohorts remains small.

The objective of this work was to study the clinical and histological presentation of patients with renal impairment during CMML, as well as their therapeutic management in a multicenter cohort. Then, we compared the hematological phenotype of patients with a kidney injury occurring during CMML with CMML patients’ controls without kidney disease.

## Methods

### Patient Selection

Patients with an established diagnosis of CMML and diagnosed with AKI, CKD, or urine abnormalities were retrospectively included. We collected data of patients from 3 different sources (Supplementary Figure S1). The first group of patients was recruited at the Brigham and Women’s Hospital, and the Massachusetts General Hospital, at Boston, MA, USA where we screened-in all consecutive patients with a diagnosis of CMML with a kidney biopsy over the past 20 years. The second one includes patients with a renal injury related to a diagnosis of vasculitis associated with CMML, from French different databases such as “Groupe Francophone des Myélodysplasies,” and “French Network of Dysimmune Disorders Associated with Hemopathies” groups.^15^ Finally, we reached out to various French scientific societies (“Société Nationale Française de Médecine Interne,” and “Société Francophone de Néphrologie Dialyse et Transplantation”) to do a retrospective collection of patient cases that fitted our inclusion criteria. We excluded every kidney disease previously known before the diagnosis of CMML, and which remained stable after the myeloid neoplasm diagnosis. As detailed in Supplementary Figure S1, the kidney diagnosis that we excluded were cases of diabetic nephropathy, membranous nephropathy associated with graft versus host disease, and hypertensive nephrosclerosis. Controls with CMML without any kidney injury were extracted from GFM prospective nationwide database.

### CMML Definitions

Diagnoses criteria for CMML were based on the World Health Organization classification revised in 2016. CMML were classified as CMML-0 (for cases with <2% blasts in the blood, and <5% blasts in the bone marrow), CMML-1 (2 to 4% blasts in the blood, and/or 5% to 9% blasts in the bone marrow), and CMML-2 (5% to 19% blasts in the blood, and 10% to 19% blasts in the bone marrow). Cytogenetic analysis and molecular findings were collected when available in the center.

### Definition of Kidney Diseases

We included adult patients (>18 years old) diagnosed with CMML, and a kidney disease less than 10 years apart. According to the National Kidney Foundation’s Kidney Disease Outcome and Quality Initiative guidelines, CKD is defined as either a decreased glomerular filtration rate (<60 ml/min per 1.73 m^2^), or markers of kidney damage: albuminuria with albumin-to-creatinine ratio ≥30mg/g, urine sediment abnormalities, electrolyte and other abnormalities due to tubular disorders, and abnormalities detected by histology among other things.^16^ Estimated glomerular filtration rate (eGFR) was calculated according to the Chronic Kidney Disease Epidemiology Collaboration formula.^17^ Kidney biopsies were locally reviewed in each pathology center.

### Clinical Data

Clinical and biological data were retrospectively collected from the patients’ charts. Clinical data included gender, age at the time of the hematological and renal diagnoses, history of hypertension, and diabetes mellitus. Extrarenal organ involvement was included if it was directly attributable to systemic manifestations of the CMML. Biological data, at the time of the hematological and renal diagnoses, included blood cell counts, serum creatinine, eGFR, urinary protein-to-creatinine ratio, and urinary sediment. Regarding underlying CMML, data collection included bone marrow cytology and cytogenetic analyses, CMML-specific prognostic scoring system, treatments, and the evolution to AML. The presence, and the type of somatic mutations by next-generation sequencing were recorded if available. At the time of the renal diagnosis, we collected antinuclear antibodies, rheumatoid factor, antineutrophil cytoplasmic antibody testing by immunofluorescence and/or enzyme-linked immunosorbent assay, serum albumin, C-reactive protein, C3/C4 levels, and the type of treatment.

### Follow-Up

During follow-up, events such as dialysis, kidney transplantation, occurrence of AML, or patient death were collected. Evaluation of kidney function with proteinuria and serum creatinine was performed at the diagnosis of CMML, at the diagnosis of the kidney injury, and at last follow-up.

### Statistics

The descriptive data of the patients were expressed in number (frequency) for the binary variables, and in median (interquartile range) for the continuous variables. Given the size of the numbers and the lack of a normal distribution of values, we used nonparametric tests. Comparisons of continuous variables between 2 groups were made using the Mann-Whitney test, and those of binary variables were made using the Chi-square test. A Kaplan-Meier method was used to estimate overall survival. The significance level was *P* < 0.05.

Statistical analyses were performed using GraphPad Prism v8.00 for MacOS (GraphPad Software, La Jolla, CA, USA), and R v4.0.3 for MacOS (R Foundation for Statistical Computing, Vienna Austria).

### Ethics

This study was conducted according to the Declaration of Helsinki and fulfilled the recommendations of the French “Commission Nationale Informatique & Libertés” CNIL. According to its policy, this study was approved by the Institutional Review Board of the Brigham and Women’s Hospital (protocol number 2019P003787), who validated a waiver of consent.

## Results

### Demographics and Hematological Characteristics

Overall, 16 patients (male gender *n* = 14, aged 76.5 years [71.9–83]) with a kidney disease associated with CMML were included in this study. CMML disease subtypes were as following: type 0 in 5 patients (25%), type 1 in 10 patients (66.7%), and type 2 in 1 patient (8.3%) (Table 1). Median monocytes count at the CMML diagnosis was 4 G/l [2.4–5], with an increase until 11 G/l [3.6–10.7] when the renal manifestations appeared. Observed molecular abnormalities were *CBL*, *RUNX1*, *SRSF2*, *IDH2* in patient FRA-01; *TET2*, *PHF6*, *ASXL1*, *SRSF2*, *JAK2* (V617F), *PTPN1*, *NRAS* in patient FRA-02; *JAK2* in patient FRA-04; *NRAS*, *KRAS*, *ASXLI1* in patient FRA-11; *EVI1* duplication in patient FRA-28; and *BCORL1*, *NRAS*, *PHF6*, *SRSF2*, *TET2*, *ZRSR2* in patient FRA-32.

**Table 1:** Hematologic and renal characteristics of 16 patients with CMML

Patients number	Gender Age (y)	Hematologic	Delay (mo)	Basal status		At renal diagnosis				Renal presentation / Renal diagnosis	Kidney pathology		Lysozyme in urine
Pt		CMML type Mutations		SCr (mg/dl)	Monocytes (G/l)	SCr (mg/dl)	uPCr (g/g)	Hu	Monocytes (G/l)		Type of interstitial infiltration	Lysozyme staining	
BWH-07	M, 76	CMML-1 NA	19	2	6	2.7	5.9	+	2.8	Nephrotic syndrome /Lysozyme nephropathy, ATN	B and T cells	+	NA
FRA-01	M, 54	CMML-1 *CBL, RUNX1, SRSF2, IDH2*	23	0.6	17	4.7	NA	NA	24	AKI /Lysozyme nephropathy	None	+	NA
FRA-02	M, 65	CMML-0 *TET2, PHF6, ASXL1, SRSF2, JAK2 (V617F), PTPN1, NRAS*	129	NA	2.6	2.3	0.4	NA	12	AKI with tubular proteinuria /Lysozyme nephropathy	None	NA	+
FRA-03	M, 72	CMML-1 NA	42	0.9	1.5	1.5	2.2	+	3.5	RPGN /CMML infiltrate	Monocytes	NA	NA
FRA-04	F, 89	NA *JAK2*	1	0.7	2.4	2.7	2	+	3	RPGN / Vasculitis with renal and skin involvements	NA	NA	NA
FRA-05	M, 78	CMML-0 NA	0	0.9	5.1	1.6	0.8	+	5.1	AKI with tubular proteinuria /CMML infiltrate	Monocytes	NA	NA
FRA-06	M, 82	CMML-1 NA	4	1.5	4.3	2.3	1.8	_	5	AKI with tubular proteinuria /CMML infiltrate, lysozyme nephropathy	Monocytes	+	NA
FRA-11	M, 65	CMML-1 *NRAS, KRAS, ASXLI1*	6	1.1	4.9	5.7	1.3	_	80	AKI with tubular proteinuria /CMML infiltrate	Monocytes	NA	NA
FRA-12	M, 74	CMML-1 NA	0	NA	2.4	HD	3.4	+	7	AKI and nephrotic syndrome /CMML infiltrate, extramedullary hematopoiesis	Immature myeloid elements	NA	NA
FRA-16	M, 81	NA	30	NA	NA	NA	NA	NA	NA	AKI with tubular proteinuria /Lysozyme nephropathy	None	+	NA
FRA-18	F, 82	NA	4	0.8	NA	1.4	NA	NA	NA	AKI / Lysozyme nephropathy	Mononuclear cells, lymphocytes	+	NA
FRA-19	M, 85	CMML-1 NA	6	NA	NA	NA	NA	_	NA	AKI with tubular proteinuria /Lysozyme nephropathy	None	NA	+
FRA-28	M, 51	CMML-2 *EVI1* duplication	0	NA	3	1	5	_	NA	Nephrotic syndrome / MCD, CMML infiltrate	Plasma cells, lymphocytes, and eosinophils	NA	NA
FRA-32	M, 74^a^	CMML-1 *BCORL1, NRAS, PHF6, SRSF2, TET2, ZRSR2*	NA	1.1	NA	1.7	NA	+	NA	Progressive CKD / Lysozyme nephropathy	T cells, plasma cells	NA	+
FRA-34	M, 90^a^	CMML-0 NA	NA	NA	1.7	2.4	NA	_	NA	AKI / Lysozyme nephropathy, CTIN	NA	NA	+
VAS-02	M, 42	CMML-0 NA	-24	NA	4	1.4	NA	_	4	AKI / Polyarteritis nodosa	NA	NA	NA

ATN, acute tubular necrosis; CKD, chronic kidney disease; CMML, chronic myelomonocytic leukemia.; CTIN, chronic tubulointerstitial nephropathy; F, female; HD, hemodialysis; Hu, hematuria; M, male; MCD, minimal change disease; Mo, months; NA, not applicable; Pt, patient; RPGN, rapidly progressive glomerulonephritis; SCr, serum creatinine; uPCr, urinary protein-to-creatinine ratio; Y, years.

a At renal diagnosis.

### Kidney and Systemic Manifestations

Time between the hematologic and kidney disease was 6 months [1.6–25.6] (Table 1). The diagnosis of CMML preceded kidney disease in 9 patients. The 2 diseases were diagnosed at the same time for 4 patients. For 1 patient, the CMML was diagnosed after kidney disease (2 missing data). One patient required hemodialysis at renal diagnosis. Median serum creatinine was 2.26 mg/dl (1.46–2.68), with an eGFR of 26 ml/min per 1.73 m^2^. Median proteinuria was 2 g/g (1.25–3.4), with a microscopic hematuria present in 6 of the 10 patients with an evaluation of the urine sediment. Leukocyturia was available in 8 patients with a positivity in 25% of the cases.

The first indication of the kidney biopsy was AKI associated to a tubular proteinuria (*n* = 9; 64%), then nephrotic syndrome (*n* = 3; 20%), rapidly progressive glomerulonephritis (*n* = 1; 7%), and a progressive CKD (*n* = 1; 7%). A preexisting CKD of unknown cause with an eGFR of 33 and 44 ml/min per 1.73 m^2^ was present in 2 patients. Hypertension was present in 82% of the patients, whereas diabetes represented 18%. Few patients had systemic symptoms (*n* = 4, 25%) with 2 organs preferentially incriminated: skin and lungs (alveolar hemorrhage and purpura). Four patients had a monoclonal immunoglobulin (3 IgG kappa and 1 IgG lambda) with a median peak of 3 g/l [1.6–9]. Median C-reactive protein was 56 mg/l [9.6–100]. Auto-immunity was detected in 5 of the 10 tested patients (antinuclear antibodies *n* = 2, antineutrophil cytoplasmic antibody with no specificity *n* = 2, and rheumatoid factor *n* = 2). One patient had a detectable cryoglobulinemia associated with a diagnosis of lysozyme nephropathy. Regarding the serum complement analyses, 25% had a low C3 and 12.5% a low C4.

### Renal Diagnoses and Histological Features of Patients With CMML

Among 16 patients, 14 (87.5%) underwent a kidney biopsy. The 2 most frequent diagnoses were lysozyme nephropathy (*n* = 9, 56%), and an interstitial infiltrate compatible with a CMML infiltration in the kidney (*n* = 6, 37.5%) (Table 1). Among the 3 patients with a nephrotic syndrome, 1 had a minimal change disease, 1 had lysozyme nephropathy with nephrotic range lysozymuria, and 1 had a renal CMML infiltrate with extramedullary hematopoiesis. One patient had both diagnoses. The features of light microscopy, immunofluorescence, and immunohistochemistry analyses of the kidney biopsies are listed in Supplementary Table S1.

Histological findings for patients with a diagnosis of lysozyme nephropathy showed lesions of acute tubular injury with focal tubular epithelial necrosis (*n* = 4), and a vacuolization of the epithelial cells of the proximal tubules (*n* = 7). Lysozyme staining was positive in 5 (*n* = 62.5%) patients (4 immunohistochemistry and 1 immunofluorescence). Four patients with lysozyme nephropathy had also lysozymuria. Interstitial fibrosis ranged from 10% to 30%. Inflammatory infiltrate with mostly mononuclear myeloid cells was described in 9 patients. For the patients who had a characterization of the infiltrate by immunohistochemistry, MPO and CD68 staining were positive (Supplementary Table S1). Regarding the 2 patients without a kidney biopsy, 1 had a diagnosis of systemic vasculitis based on the skin biopsy showing C3 deposits along the capillary of the superficial derma, with a renal presentation associating AKI and glomerular proteinuria. The other patient had polyarteritis nodosa with renal microaneurysms.

### Outcomes

At renal diagnosis, 3 patients were already treated for their CMML by hydroxyurea (Table 2). After the renal diagnosis, hematologic treatment was started or modified in 6 patients (37.5%). It consisted of the introduction of 1 or several of the following treatments: hydroxyurea, azacitidine, mercaptopurine, and an allogeneic stem cell transplantation. Three patients had a diagnosis of CMML concomitant to or less than 2 months after renal involvement. Each one received a different hematologic treatment: hydroxyurea, azacytidine, and decitabine. Four patients received steroids after the renal diagnosis. One of them was diagnosed with polyarteritis nodosa and his treatment consisted of cyclophosphamide, azathioprine, and steroids.

**Table 2:** Outcomes of 16 patients with CMML and renal involvement

Patients number	Hematologic treatments		Follow-up (mo)	Renal	Outcomes			
Malignancy	At diagnosis of the kidney disease	After		Diagnosis	eGFR at last follow-up	GFR change	Malignancy	Overall
CMML-1	Hydroxyurea	Hydroxyurea	9.9	LyN	25	2	CMML-2	Alive
CMML-1	Hydroxyurea	Allogeneic SCT	10.9	LyN	50	37	CR	Alive
CMML-0	No	Hydroxyurea	32.2	LyN	24	24	CMML-2	Death
CMML-1	No	Azacitidine	24.7	CMMLi	49	2	Stable	Alive
CMML	No	Hydroxyurea Steroids	34.9	Vasculitis	42	22	Stable	Alive
CMML-0	No	Azacitidine Steroids	4.3	CMMLi	25	-15	AML	Alive
CMML-1	No	NA	0.7	CMMLi LyN	NA	NA	NA	Death
CMML-1	Hydroxyurea	Hydroxyurea Azacitidine Steroids	36.6	CMMLi	Dialysis	46	Stable	Death
CMML-1	No	Decitabine	9.5	CMMLi	80	80^a^	Failure	Death
CMML	NA	NA	NA	LyN	NA	NA	NA	Death
CMML	NA	NA	NA	LyN	NA	NA	NA	NA
CMML-1	NA	NA	NA	LyN	NA	NA	NA	NA
CMML-2	No	Hydroxyurea mercaptopurine	14.8	MCD CMMLi	86	−1	Failure	Alive
CMML-1	No	Patient refusal	68	LyN	34	−4	Stable	Alive
CMML	No	Hydroxyurea	15.3	LyN	25	2	AML	Alive
CMML-0	No	Cyclophosphamide, steroids, azathioprine	39.5	PAN	NA	NA	Stable	Alive

AML, acute myeloid leukemia; CMML, chronic myelomonocytic leukemia; CMMLi, CMML infiltrate; CR, complete remission; eGFR, estimated glomerular filtration rate; HI, hematologic improvement; LyN, lysozyme nephropathy; MCD, minimal change disease; NA, not applicable; PAN, polyarteritis nodosa; SCT, stem cell transplantation.

a Hemodialysis at renal diagnosis.

After a median follow-up of 15 months (9.9–34.9), hematologic malignancy worsened in 6 patients (2 CMML-2, 2 AML, and 2 failure), with 5 deaths at last follow-up (survival rate 64.3%, 2 missing data). After treatment, kidney function worsened in 1 (9%) patient. It was stable (eGFR change ± 5 ml/min per 1.73 m^2^) for 5 (45.5%) patients and got improved in 5 (45.5%) patients (4 missing data). One patient refused to be treated by azacitidine, and his kidney function remained stable after 68 months.

### Comparison Between CMML With and Without Kidney Injuries

**Figure 1:**
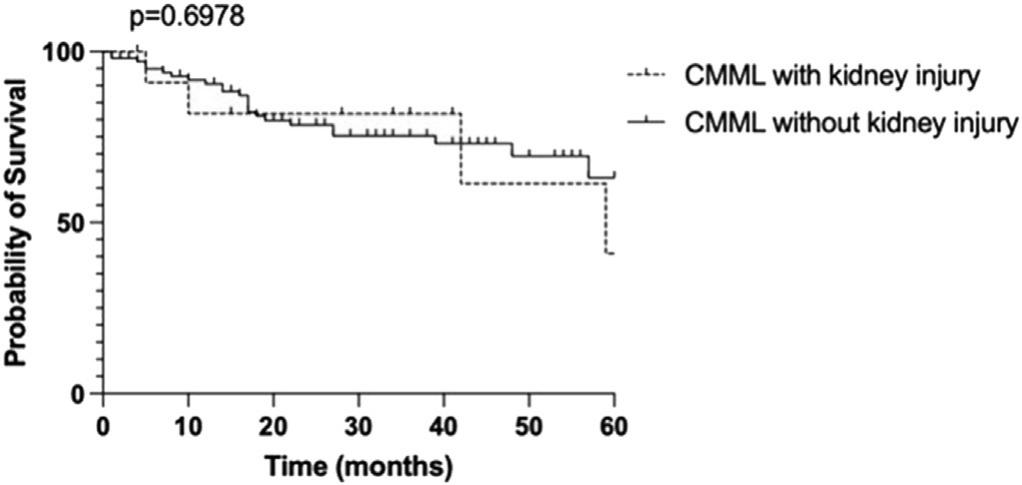
Kaplan Meier curve for the overall survival according to CMML with and without kidney injury. CMML, chronic myelomonocytic leukemia.

Compared to 116 patients with CMML without a kidney injury, patients with a CMML associated with a kidney involvement had a higher monocyte count (1.8 G/l [1.3–2.8] vs. 5 G/l [3.6–10.8], respectively, *P* < 0.001) (Table 3). Moreover, the main subtype of CMML in the group with a kidney injury was the CMML-1 (66.7% vs. 25% in the control group, *P* = 0.005). Even if there was no difference in terms of prognostics, with an International Prognosis Scoring System-Revised above 3.5 in 28.4% of CMML control group against 20% (*P* = 1), patients with CMML with a kidney injury were more susceptible to develop an AML: 30.8% versus 6.9% in the control group (*P* = 0.02). Patients with CMML who had a kidney injury were more eligible to receive a specific hematologic treatment, with hydroxyurea, or hypomethylating agents (84.6% and 19.8% in the control CMML group, *P* < 0.001). The median follow-up from CMML diagnosis was 21 months [7–39.5] in patients without a kidney injury, and 34.9 months [13.9–46.5] in patients with a kidney injury (*P* = 0.174). The median survival was 59 months, and not reached for the follow-up period in CMML with and without a kidney injury, respectively (*P* = 0.6978, odds ratio: 1.18 [95% confidence interval 0.43–3.28]) (Figure 1).

**Table 3:** Characteristics of CMML according to the presence/absence of a kidney injury

Variable	Total (*n* = 132)	CMML without kidney injury (*n* = 116)	CMML with kidney injury (*n* = 16)^a^	*P*-value
Age (yrs)	77 [70–82]	77 [70–82]	75 [65–82]	0.359
Male	87 (65.9%)	73 (62.9)	14 (87.5)	0.088
CMML type				
CMML-0	70 (54.7)	67 (57.8)	3 (25)	0.036
CMML-1	37 (28.9)	29 (25)	8 (66.7)	0.005
CMML-2	21 (16.4)	20 (17.2)	1 (8.3)	0.689
Dysplasia lineage number				
0	63 (54.3)	63 (58.3)	0 (0)	0.001
1	29 (25)	25 (23.1)	4 (50)	0.106
2	20 (17.2)	17 (15.7)	3 (37.5)	0.139
3	4 (3.4)	3 (2.8)	1 (12.5)	0.252
Blasts (%)	4 [2–7]	4 [2–7]	5 [3–10]	0.339
Abnormal karyotype	33 (26.6)	31 (26.7)	2 (25)	1.000
Neutrophils count (G/l)	5 [3–9]	5 [3–8.6]	5.9 [3.5–26.6]	0.297
Monocytes count (G/l)	1.9 [1.4–3.1]	1.8 [1.3–2.8]	5 [3.6–10.8]	<0.001
Hemoglobin (g/dl)	11.5 [9.7–13.2]	11.5 [9.9–13.3]	10.1 [9.2–11.6]	0.072
Platelets (G/l)	145 [85–222]	137 [77–218]	202 [149–267]	0.104
IPSS-R	3 [2–4]	3 [2–4]	3 [2.7–3.3]	0.734
IPSS-R > 3.5	34 (28.1)	33 (28.4)	1 (20)	1.000
Specific hematologic treatment	34 (26.4)	23 (19.8)	11 (84.6)	<0.001
Progression to AML	12 (9.3)	8 (6.9)	4 (30.8)	0.020
Time between CMML and AML (mo)	22.5 [12.3–29]	22.5 [12.7–27]	86 [45–127]	1.000
Follow-up (mo)	22 [7.8–41.5]	21 [7–39.5]	34.9 [13.9–46.5]	0.174

AML, acute myeloid leukemia; CMML, chronic myelomonocytic leukemia; IPSS-R, International Prognosis Scoring System-Revised.

a Missing data.

## Discussion

CMML is a myeloid neoplasm characterized as a myelodysplastic syndrome/ myeloproliferative neoplasm overlap syndrome. CMML is associated with significant monocytosis, which is correlated with AKI and CKD.^8^

In our study, the time between the diagnosis of CMML and the development of a kidney injury was 6 months, which is close to the delay reported by Grignano *et al.*^5^ in their study describing autoimmune and inflammatory diseases associated with CMML. However, Belliere *et al.*^13^ identified a delay of 7.7 ± 2 years for CMML or BCR-ABL-negative myeloproliferative neoplasms patients. We found that lysozyme nephropathy and an infiltration by the CMML were by far the most frequent kidney diseases associated with CMML. This was similar to the results of Gipe *et al.*^18^ who described the spectrum of renal pathological findings among 14 patients with CMML. In large cohorts of autoimmune and inflammatory diseases associated with CMML, systemic vasculitis was the main related feature.^5^^,^^6^ Despite this fact, we onlyhad 2 cases of vasculitis in our cohort. This contradiction can be explained in part by the pathophysiology of the specific kidney injuries associated with CMML.

Lysozyme is a small cationic protein produced by monocytes, that is freely filtered by the glomerular filtration barrier, and reabsorbed by proximal tubules.^12^ An overproduction of lysozyme can be observed in CMML. Above a certain threshold, lysozyme becomes toxic to proximal tubular cells, leading to acute tubular injury. A kidney biopsy is needed to establish the diagnosis of lysozyme nephropathy, because elevated serum, and urine lysozyme levels commonly occur in patients with CMML, without being associated with the development of AKI.^19^ Lysozyme-induced nephropathy is characterized by acute tubular injury with abundant cytoplasmic granular inclusions that stain strongly for lysozyme.^12^ Importantly, lysozyme-induced nephropathy can respond to renal supportive care and cytorectuction, arguing for a monitoring of lysozymuria in patients with CMML, and if necessary a renal biopsy to confirm the diagnosis.^18^

Kidney infiltration by CMML is mostly seen in the tubulointerstitial compartment, even if it has already been described in the glomerulus.^13^^,^^20^^,^^21^ Two types of infiltrates have been characterized by immunohistochemistry: extra-medullary hematopoiesis, with the presence of megakaryocytes (positive CD61 marker); and a myelomonocytic infiltrate (positive MPO and CD68 markers).^13^^,^^20^ However, it can be difficult to properly assess the percentage of renal parenchymal infiltration by myelomonocytic cells, because the nature of the infiltration can be patchy with a lot of inflammation. Further work assessing why the kidney is a target of the myelomonocytic infiltration are also needed. It has been shown that the presence of clonal hematopoiesis in bone marrow was also present in cutaneous immature myeloid cell infiltrate in a new entity called myelodysplasia cutis, because the same mutations were found in both tissues.^22^ This suggests that clonal monocytes could also participate in kidney lesions, with different hypothetical mechanisms involved such as chemokine gradient, kidney as a proliferation niche.

At last follow-up, kidney function evolution was heterogenous, with 4 patients evolving to CKD 3, 4 patients to CKD 4, and 1 patient to end-stage kidney disease, which was comparable to Belliere *et al.*^13^ Interestingly, in Gipe *et al.* ^8^ study, although supportive care and cytoreductive therapy did help improve renal function in all patients with lysozyme-induced nephropathy, they continued to have CKD, potentially arguing for earlier cytoreduction and renal risk factor mitigation. Compared to CMML controls, patients with CMML who had a kidney injury were more eligible to receive a specific hematologic treatment. CMML associated with a kidney involvement seemed to have worse baseline prognostic factors (monocytes count, type of CMML, number of dysplasia, and evolution toward AML), but we did not observe a statistically significant difference in terms of overall survival.

Limitations of this work are mainly due to its retrospective design, and the small sample size of the cohort. To push further the analysis, the next objectives will be to collect kidney biopsy samples to further characterize the pathological features of the kidney injuries (infiltrate and lysozyme staining) associated with CMML. One of our perspectives will be to realize next-generation sequencing on the kidney biopsies of patients with an infiltrate, to see if we can find a significant renal clonal infiltrate of CMML cells.

In conclusion, a regular screening for blood pressure, proteinuria, and kidney function should be proposed to all patients with CMML. In case of kidney dysfunction, kidney biopsy seems important to confirm the diagnosis in patients with CMML or to rule out differential diagnoses. Dosage of lysozymuria can also be informative, although not easily accessible in routine. In this cohort of patients with CMML with a kidney injury, the 2 most frequent kidney complications were lysozyme-induced nephropathy, and kidney infiltration by the CMML. The occurrence of a kidney injury in the course of a CMML seems to worsen the prognosis of these patients.

## Appendix

### List of the MINHEMON, GFM and French VEXAS Group

*List of the MINHEMON group:* Marie-Camille Lafargue, Mickaël Bobot, Thibault Comont, Nathalie Cambier, Kamel Laribi, Olivier Fain,Lionel Adès, Pierre Fenaux, Arsène Mekinian

*List of GFM group:* Thibault Comont, Nathalie Cambier, Kamel Laribi, Olivier Fain, Lionel Adès, Pierre Fenaux, Arsène Mekinian

*List of French VEXAS group:* Thibault Comont, Olivier Fain, Lionel Adès, Pierre Fenaux, Arsène Mekinian

## Disclosure

AM is investigator of CELGENE, ROCHE, CHUGAI founded trials with APHP and Hopital 15-20 promotion; AM received several fees for congress travels and experts’ use from LFB, SANOFI, SHIRE, and CELGENE. All other authors have declared no conflicting interest.

## Supplementary Material

Supplementary File (PDF)

**Figure S1.** Flowchart.

**Table S1.** Renal pathology of 14 patients with a kidney injury associated to a chronic myelomonocytic leukemia.
