# Supplementary material for: Chronic Myelomonocytic Leukemia Patients With Lysozyme Nephropathy and Renal Infiltration Display Markers of Severe Disease
Source: Kidney Int Rep. 2023 Sep 7;8(12):2733–41. doi: 10.1016/j.ekir.2023.09.005 (PMC10719588; doi:10.1016/j.ekir.2023.09.005)
Supplement: Supplementary File (PDF) [file mmc1.pdf]

Supplementary: Table S1: Renal pathology of 14 patients with a kidney injury associated to a CMML

Patients	Glomeruli	Tubules	Interstitium	Vessels	IHC	IF	Diagnosis
**BWH-07**	48 (12 sclerotic). Mild mesangial expansion and signs of remodeling of the capillary walls with frequent double contours. Global (25%) and segmental (10%) glomerulosclerosis.	Severe acute tubular injury, with focal tubular epithelial necrosis and prominent lysozyme-positive granules in the tubular epithelium.	Mild to moderate interstitial inflammation reactive for immunoperoxidase stains for B- and T-cell markers.	Severe arterial and arteriolar sclerosis.	Tubules that reveal prominent eosinophilic granules that reveal positive immunoperoxidase reaction to lysozyme. Interstitial inflammation reactive for immunoperoxidase stains for B- and T-cell markers.	Intense fibrin deposition in the interstitium.	Lysozyme nephropathy, CTIN
**FRA-01**	15 (0 sclerotic). Segmental glomerulosclerosis.	Cytoplasmic vacuolization of the proximal epithelial cells.	Mild interstitial fibrosis (<10%).	Mild arteriolar sclerosis.	Tubules with positive immunoperoxidase reaction to lysozyme.	No significant deposits.	Lysozyme nephropathy
**FRA-02**	12 (1 sclerotic). Optically normal glomeruli.	Vacuolization of the proximal epithelial cells.	Interstitial fibrosis (20%) with mild inflammation.	Moderate arterial and arteriolar sclerosis.	NA	No significant deposits.	Lysozyme nephropathy
**FRA-03**	6 (1 sclerotic). Optically normal glomeruli.	No tubular atrophy, no ATN.	Inflammatory interstitial infiltrate with mononuclear, myeloid cells.	No arterial, nor arteriolar sclerosis.	Interstitial infiltrate: CD45+/-, CD34-, MPO+, focal GLUT1+, CD68+, CD56- with CD138+ plasma cells, and rare B, and T lymphocytes.	No significant deposits.	CMML infiltrate
**FRA-05**	13 (1 sclerotic). Optically normal glomeruli.	ATN	Interstitial fibrosis. Diffuse monocytic cell infiltrate.	Moderate arteriolar sclerosis.	NA	No significant deposits.	CMML infiltrate
**FRA-06**	42 (1 sclerotic). Irregular thickening of the Bowman's capsule. No proliferation, nor deposits.	Acute tubular injury with hyaline cylinders and cytoplasmic resorption granules.	Interstitial fibrosis. Interstitial infiltrate with monocytic and polynuclear cells.	Moderate arterial, severe arteriolar sclerosis.	Interstitial infiltrate: CD68+, CD15+, MPO+, CD34-.	Lysozyme (++) for the tubular granules.	CMML infiltrate, lysozyme nephropathy

**FRA-11**	9 (1 sclerotic).<br>Mild ischemic glomeruli.	NA	Diffuse interstitial infiltrate with monocytic cells.	No arteriolar sclerosis.	Interstitial infiltrate: MPO+, CD15-, CD34- and rare B lymphocytes (CD20+), T lymphocytes (CD3+).	No significant deposits.	CMML infiltrate
**FRA-12**	25 (5 sclerotic).<br>Segmental and global mesangial hyperplasia.<br>Neutrophils in the capillary lumen.	ATN.	Polymorphic interstitial infiltrate (15-20%)	Mild arterial sclerosis.	Immature myeloid elements.	No significant deposits.	CMML infiltrate
**FRA-16**	21 (8 sclerotic).	Acute tubular injury with vacuolization of the epithelial cells.	Interstitial fibrosis (25%)	Moderate arterial sclerosis.	Acute tubular alterations with lysosomal overload.	No significant deposits.	Lysozyme nephropathy
**FRA-18**	28 (9 sclerotic).<br>Optically normal glomeruli.	Vacuolization of the proximal epithelial cells with hyaline droplets.	Interstitial fibrosis (20%).<br>Interstitial infiltrate with mononuclear cells with lymphocytes and rare plasma cells.	Moderate arterial and mild arteriolar sclerosis.	Tubules with positive immunoperoxidase reaction to lysozyme.	No significant deposits.	Lysozyme nephropathy
**FRA-19**	8 (0 sclerotic).<br>Optically normal glomeruli.	Vacuolization of the proximal epithelial cells.	Interstitial fibrosis (10-15%).	Severe arterial and arteriolar sclerosis.	NA	No significant deposits.	Lysozyme nephropathy
**FRA-28**	13 (1 sclerotic).<br>Optically normal glomeruli.	Acute tubular injury.	Polymorphic interstitial infiltrate with plasma cells, lymphocytes, and eosinophils.	Normal vessels	NA	No significant deposits.	MCD, CMML infiltrate
**FRA-32**	NA	ATN.<br>Tubular atrophy (30%).<br>Vacuolization of the epithelial cells.	Interstitial fibrosis (30%).<br>Inflammatory interstitial infiltrate with lymphocytes and plasma cells.	NA	Interstitial infiltrate with predominant T lymphocytes.	NA	Lysozyme nephropathy
**FRA-34**	NA	NA	NA	NA	NA	NA	NA

Abbreviations: ATN: acute tubular necrosis. CMML: chronic myelomonocytic leukemia. CTIN: chronic tubulointerstitial nephropathy. IF: immunofluorescence. IHC: immunohistochemistry. MCD : minimal change disease. NA: not applicable.

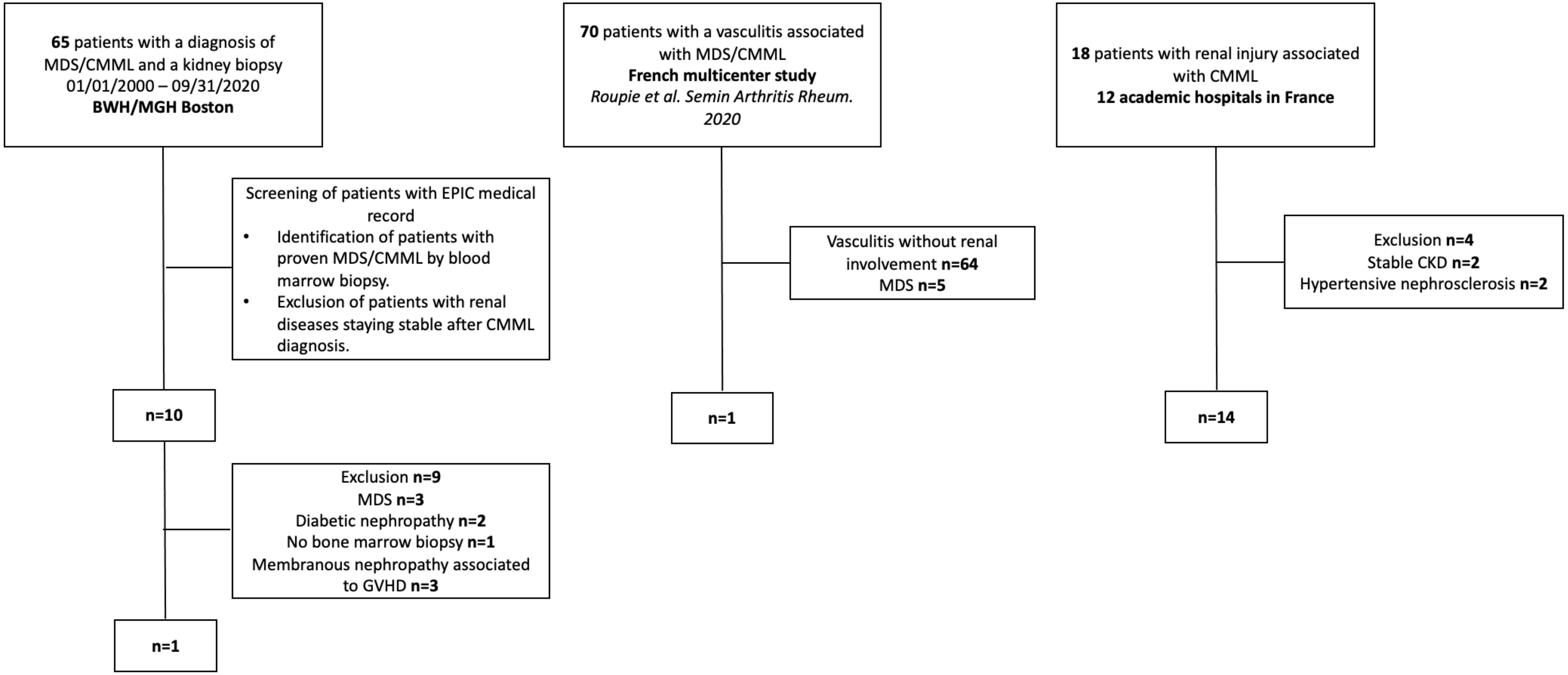

Supplementary Figure S1: *flowchart. BWH: Brigham and Women's Hospital. CKD: chronic kidney disease. CMML: chronic myelomonocytic leukemia. GVHD: graft versus host disease. MDS: myelodysplastic syndromes. MGH: Massachusetts General Hospital*
